# Defining polypharmacy in older adults: a cross-sectional comparison of prevalence estimates calculated according to active ingredient and unique product counts

**DOI:** 10.1007/s11096-025-01882-7

**Published:** 2025-02-15

**Authors:** Georgie B. Lee, Sarah M. Hosking, Christopher Etherton-Beer, Julie A. Pasco, Lana J. Williams, Kara L. Holloway-Kew, Amy T. Page

**Affiliations:** 1 School of Medicine, IMPACT - Institute for Mental and Physical Health and Clinical Translation, Deakin University, Geelong, VIC Australia; 2 WA Centre for Health and Ageing, University of Western Australia, Crawley, WA Australia; 3 Department of Medicine-Western Health, The University of Melbourne, St Albans, VIC Australia; 4 Barwon Health, Geelong, VIC Australia; 5 Department of Epidemiology and Preventive Medicine, Monash University, Melbourne, VIC Australia

**Keywords:** Aging, Epidemiologic methods, Inappropriate prescribing, Polypharmacy, Prevalence

## Abstract

**Background:**

Polypharmacy is common in older adults and may be associated with poor outcomes. However, methods used to define polypharmacy are rarely reported precisely, with potential implications for polypharmacy exposure estimates.

**Aim:**

The aim was to investigate prevalence estimates according to different methods in an Australian population-based sample of older adults.

**Method:**

This cross-sectional study included 735 adults aged ≥ 60 years participating in the Geelong Osteoporosis Study. Current prescription, non-prescription and complementary and alternative medicines were self-reported. Counting methods included the number of active ingredients and unique products. Polypharmacy and hyperpolypharmacy were determined using ≥ 5 and ≥ 10 medicine cut points respectively. Prevalence was estimated using ingredient- and product-level counts according to criteria defined by medicine schedule and type (i.e. scheduled prescription, non-prescription). Non-parametric testing measured differences between counting methods, univariate logistic regressions investigated disagreement between total counts and polypharmacy exposure.

**Results:**

Polypharmacy prevalence (scheduled prescription medicines) was 30.3% and 35.9% (products versus ingredients). Prevalence increased to 52.8% and 57.3% when counts considered any medicine. Adults aged ≥ 80 years were most likely to use prescription combination products (OR 2.22 [95% CI 1.46, 3.35] *p* < 0.01), however, age was not associated with disagreement between product and ingredient polypharmacy exposure. Being male was associated with both prescription combination product use (OR 1.79 [95% CI 1.29, 2.47] *p* < 0.001) and disagreement between polypharmacy exposures (OR 2.29 [95% CI 1.15, 4.47] *p*=0.02).

**Conclusion:**

Polypharmacy prevalence estimates varied substantially depending on the method applied. These data indicate the need for standardised reporting specific to medicines data and polypharmacy measures.

**Supplementary Information:**

The online version contains supplementary material available at 10.1007/s11096-025-01882-7.

## Impact statements

- Polypharmacy prevalence estimates varied by up to 27% between different methods, with implications for who may be classified as exposed or not. This suggests that published research without clearly defined counting methods and inclusion criteria may not be easily interpreted or compared.
- The substantial variability observed between measures of medicine use demonstrates a clear need for the development of a minimum methods reporting standard for research that counts medicines, aiming to strengthen the evidence base and better target interventions aimed at reducing preventable medicine-related harm.

## Introduction

The use of multiple medicines to manage symptoms and prevent complications, known as polypharmacy, is common in older adults [1]. While medicines can provide benefits, they may also cause significant adverse effects [2]. Research consistently shows that multi-medicine regimens are linked to poor clinical outcomes among older adults [3, 4]. Consequently, polypharmacy is monitored as a quality indicator in various settings, including aged care facilities, however, these measures lack comparative value if polypharmacy is not defined consistently. While the interpretation, comparison, and reproducibility of polypharmacy research is a challenge when methods are not reported precisely.

Polypharmacy definitions vary across the literature. The concurrent use of ≥ 5 scheduled medicines is the most common [5, 6]; similarly, the term hyperpolypharmacy often refers to ≥ 10 medicines [3, 4]. However, the cut point applied may vary by the study’s aims and setting. In the context of high-risk prescribing, such as in prescribing cascades [7] or the concurrent use of medicines with clinically significant drug-drug interactions [8], polypharmacy may be as few as two medicines. Polypharmacy may also be based on implicit criteria, such as taking more medicines than clinically indicated [9], which are important for determining the clinical appropriateness of polypharmacy. However, broader numeric definitions are often used to stratify risk to regulate access to services, such as medication reviews, or to monitor and profile the likelihood of adverse events at a population level [10, 11].

Beyond the cut point, polypharmacy definitions are often oversimplified without detail on the types of medicines captured. Measures may count prescription-only medicines [12] or include non-prescription medicines (i.e. over-the-counter medicines, available without a prescription) [13]. Likewise, counts may capture complementary and alternative medicines (CAM) use, such as herbal medicines and dietary supplements [14]. Data availability is often a key factor in which polypharmacy definition is applied, however, in many cases, the medicines categories included are not described [6]. Additional details that may be important when defining polypharmacy include the administration schedule and the route forms captured. Studies may capture scheduled medicines taken regularly [3], some include unscheduled medicines only used as required (prn) [4], while others do not discriminate [15]. Likewise with administration route, some definitions only count oral forms [14], or medicines with a systemic action [16], though this level of detail is rarely published.

Estimates of polypharmacy prevalence may also vary depending on how combination products are handled. The medicine count for products containing a single active ingredient are often stable across methods that consider the number of active ingredients and unique products used. However, some consumers are prescribed combination products containing ≥ 2 active ingredients, a practice aimed improving reducing medicine burden to improve adherence. Both product and ingredient counts are used in research [13, 14] and are valid methods, however, details regarding the unit of measure are often omitted [4, 17–19]. For people using combination products, their total medicine counts will likely vary between the methods, with potential implications for polypharmacy exposure status.

Current polypharmacy research does not report polypharmacy using consistent criteria [5, 6], which is problematic for determining how exposure is classified, limiting the interpretation, comparison and reproducibility of findings.

### Aim

To compare polypharmacy prevalence estimates according to calculations based on active ingredient and unique product counts in an Australian population-based sample of older adults.

### Ethics approval

Written informed consent was provided by all participants. Approval for the GOS and secondary analysis of the current study were obtained from the Barwon Health Human Research Ethics Committee [92/01 and 00/56].

## Method

### Population

This cross-sectional study utilised data from the 15-year follow-up (2011–2014 for women and 2018–2020 for men) of the Geelong Osteoporosis Study (GOS), a longitudinal, population-based health and lifestyle study, in south-eastern Australia [20]. A detailed description of the study population is available elsewhere [20]. In brief, age-stratified samples of adults were randomly selected from electoral rolls in the Barwon Statistical Division (BSD), and participants were followed up periodically from 1993 for women and 2001 for men [20]. For this study, data were obtained for those aged ≥ 60 years who participated in the 15-year follow-up (n = 744) and provided data on current medicines (n = 736). One participant was excluded due to insufficient detail, resulting in a sample of 735 adults: 371 women and 364 men.

### Definitions

Current medicine use was collected via self-report either at the time of the participant’s clinical appointment or via questionnaire. Participants were urged to bring medicines or medicines lists to their clinical appointment to be cross-checked against their self-report or recorded by a researcher. They were asked to report all current medicines, including prescription and non-prescription medicines, and any vitamins, minerals, herbs or digestive aids. Medicine name, dose, start date, and reasons for taking the medicine were also recorded, no other prompts were provided.

Self-reported medicines data were standardised using the Anatomic Therapeutic Chemical (ATC) system and coded according to each active ingredient listed in each pharmacological preparation [21]. All medicines were coded and assigned to categories using the clinical judgement of a pharmacist researcher (ATP). Categories included medicine type (i.e. prescription, non-prescription, or CAM), administration route (oral or other) and administration frequency using the dose data collected during self-report by participants (i.e. scheduled, *pro re nata* (prn [as required]) or short-course). When medicines could be classified into more than one category (i.e. vitamin d as prescription/non-prescription/CAM; salbutamol as prescription/non-prescription), they were coded according to their most common usage. Antibiotics were assumed to be short-course unless participants specifically indicated otherwise. Self-reported vaccines were excluded from the analysis (n = 1). Reported medicines without enough information to accurately categorise administration frequency (n = 142) were included in the any medicines counts only. To capture the number of unique products used, each active ingredient was counted fractionally according to the number of actives in each preparation. For example, a combination ear ointment contains four active ingredients, neomycin, gramicidin, nystatin and triamcinolone, each individually coded as 0.25 products, equalling one unique product when summed. Combination CAM products containing > 4 active components, such as multivitamins, were coded as a single-ingredient preparation.

Polypharmacy was defined as ≥ 5 and hyperpolypharmacy was ≥ 10 medicines, according to active ingredients and product count [10]. Various inclusion criteria were applied, i.e. scheduled prescription medicines only, the addition of non-prescription, and CAMs. An all-encompassing measure of any medicine also included unscheduled and uncategorised medicines. Medicines of all route forms were counted. Total medicines were coded as binary variables (i.e. polypharmacy absent/present) across the methods.

Agreement and disagreement between counting methods were defined according to whether an individual’s total ingredient and product count was the same (agreement) or different (disagreement). Likewise, disagreement between ingredient and product polypharmacy was indicated when exposure status was different between the measures. In our sample, participants with prevalent combination product use (≥ 1 product with multiple active ingredients) showed 100% disagreement between the total number of active ingredients and unique products, while those using no combinations had 100% agreement between counts (Table S1). Thus, combination medicine use was considered an indicator of this disagreement.

### Data analysis

Data were analysed using Stata/MP 18.0 (StataCorp LLC, Texas USA). The number and proportion of participants exposed to polypharmacy were determined according to each binary cut-point and medicine-type inclusion criteria. Mean and standard deviation (SD) were reported for clearly normally distributed data; otherwise, median and interquartile range (IQR) were also included for completeness, while medicine range offers a comprehensive description of usage in the population.

To investigate the difference between various measures, a significance level at α = 0.05 was set. Median medicine use by active ingredient and unique product counts were compared using the Wilcoxon matched-pairs signed-rank test to calculate p values. The asymptotic McNamar test was used to measure the difference between active ingredient and unique product polypharmacy prevalence. Relative percentage difference was calculated with active ingredients as the reference measure. Univariate logistic regressions investigated how participant characteristics differed between those who used ≥1 combination products and those who used only single-ingredient products. Univariate logistic regression was also used in a subpopulation analysis of people with active ingredient polypharmacy, investigating associations with disagreement in polypharmacy exposure status, i.e. participants with ≥ 5 active ingredients (exposed) but < 5 unique products (unexposed).

## Results

Of the 735 older adults included in the study, the median age was 71 years (IQR 65–78) and 50.5% (n = 371) were female (Table 1). Self-reported medicine use was common, with 694 (94.4%) participants listing at least one medicine of any kind (Table S2). Scheduled prescription medicines were reported by 86.1% (n = 633) of participants, with a median number of 3 for both active ingredients (IQR 1–6), and unique prescription products (IQR 1–5). Fewer participants reported using scheduled non-prescription medicines (n = 232 [31.6%] and CAMs (n = 293 [39.9%]) and the median count was 0 (IQR 0–1) for both active ingredient and unique products and both medicine categories (Table S2). Despite comparable distribution, the difference between the product and ingredient counting methods was significant across the various inclusion criteria, excluding short-course medicines (Table S2).

**Table 1:** Participant characteristics (n = 735)

	n (%)
Age	
Median age (IQR)	71 years (65–78 years)
60–69 years	333 (45.3%)
70–79 years	252 (34.3%)
≥ 80 years	150 (20.4%)
Sex	
Female	371 (50.5%)
Male	364 (49.5%)

The use of any combination medicine was observed in 37.8% (n = 278) of participants, with 28.4% (n = 209) of the sample using ≥ 1 scheduled prescription combination (Table S3). Scheduled combination non-prescription and CAMs were less common (n = 34 [4.4%]; n = 30 [4.1%]) (Table S1). Adults in the oldest age group (≥ 80 years) were most likely to use scheduled combination prescription (OR 2.22 [95% CI 1.46, 3.35] *p* < 0.001) and non-prescription medicines (OR 2.85 [95% CI 1.15, 7.03] *p* < 0.02) (Table S3). Compared to women, men were more likely to use prescription combinations (OR 1.79 [95% CI 1.29, 2.47] *p* < 0.001) but less likely to use non-prescription combination products (OR 0.32 [95% CI 0.14, 0.73] *p* 0.01) (Table S3).

### Polypharmacy definitions

Table 2 summarises polypharmacy and hyperpolypharmacy prevalence estimates according to active ingredient and unique product counts, by inclusion criteria in total and stratified by sex and age. Prescription-only polypharmacy prevalence was 35.9%, according to active ingredients, and 30.3%, using product counts (Table 2). Of the 264 participants identified as exposed to active ingredient polypharmacy, 41 (15.5%) were classified as unexposed to unique product polypharmacy (Table 2). Of these, the probability of disagreement was greatest among males (OR 2.29 [95% CI 1.15, 4.47] *p* 0.02), with no significant association with age (Table 3).

**Table 2:** Difference in polypharmacy prevalence according to active ingredient and product counts by inclusion criteria, age and sex

	Polypharmacy prevalence		Difference		
	Active ingredients n (%)	Product count n (%)	Absolute difference n (%)	Relative percentage difference~	*P* value^
*Polypharmacy (5 or more medicines)*					
Scheduled prescription					
Total cohort	264 (35.9)	223 (30.3)	41 (5.6)	15.5%	< 0.001
Males	122 (33.5)	96 (26.4)	26 (7.1)	21.3%	< 0.001
Females	142 (38.3)	127 (34.2)	15 (4.0)	10.6%	< 0.001
60–69 years	74 (22.2)	62 (18.6)	12 (3.6)	16.2%	< 0.001
70–79 years	108 (42.9)	96 (38.1)	12 (4.8)	11.1%	< 0.001
80 + years	82 (54.7)	65 (43.3)	17 (11.3)	20.7%	< 0.001
Scheduled prescription and non-prescription medicines					
Total cohort	311 (42.3)	274 (37.3)	37 (5.0)	11.9%	< 0.001
Males	139 (38.2)	118 (32.4)	21 (5.8)	15.1%	< 0.001
Females	172 (46.4)	156 (42.0)	16 (4.3)	9.3%	< 0.001
60–69 years	89 (26.7)	76 (22.8)	13 (3.9)	14.6%	< 0.001
70–79 years	127 (50.4)	113 (44.8)	14 (5.6)	11.0%	< 0.001
80 + years	95 (64.4)	85 (56.7)	10 (6.7)	10.5%	< 0.01
Scheduled prescription, non-prescription and CAM					
Total cohort	371 (50.5)	341 (46.4)	30 (4.1)	8.1%	< 0.001
Males	165 (45.3)	148 (40.7)	17 (4.7)	10.3%	< 0.001
Females	206 (55.5)	194 (52.0)	13 (3.5)	6.3%	< 0.001
60–69 years	122 (36.6)	108 (32.4)	14 (4.2)	11.5%	< 0.001
70–79 years	144 (57.1)	133 (52.8)	11 (4.4)	7.6%	< 0.001
80 + years	105 (70.0)	100 (66.7)	5 (3.3)	4.8%	0.02
Any medicine*					
Total cohort	421 (57.3)	388 (52.8)	33 (4.5)	7.8%	< 0.001
Males	180 (49.4)	163 (44.8)	17 (4.7)	9.4%	< 0.001
Females	241 (65.0)	225 (60.6)	16 (4.3)	6.6%	< 0.001
60–69 years	149 (44.7)	134 (40.2)	15 (4.5)	10.1%	< 0.001
70–79 years	156 (61.9)	144 (57.1)	12 (4.8)	7.7%	< 0.001
80 + years	116 (77.3)	110 (73.3)	6 (4.0)	5.2%	0.01
*Hyperpolypharmacy (10 or more medicines)*					
Scheduled prescription					
Total cohort	32 (4.4)	17 (2.3)	15 (2.0)	47%	< 0.001
Males	19 (5.2)	10 (2.8)	9 (2.5)	47%	< 0.01
Females	13 (3.5)	7 (1.9)	6 (1.6)	46%	0.01
60–69 years	2 (0.6)	0 (0)	2 (0.6)	100%	0.16
70–79 years	15 (6)	6 (2.4)	9 (3.6)	60%	< 0.01
80 + years	15 (10)	11 (7.3)	4 (2.7)	27%	0.04
Scheduled prescription and non-prescription medicines					
Total cohort	47 (6.4)	30 (4.1)	17 (2.3)	36%	< 0.001
Males	25 (6.9)	15 (4.1)	10 (2.7)	40%	< 0.01
Females	22 (5.9)	15 (4)	7 (1.9)	32%	< 0.01
60–69 years	4 (1.2)	0 (0)	4 (1.2)	100%	0.04
70–79 years	23 (9.1)	16 (6.4)	7 (2.8)	30%	< 0.01
80 + years	20 (13.3)	14 (9.3)	6 (4.0)	30%	0.01
Scheduled prescription, non-prescription and CAM					
Total cohort	77 (10.5)	56 (7.6)	21 (2.9)	27%	< 0.001
Males	41 (11.3)	28 (7.7)	13 (3.6)	32%	< 0.001
Females	36 (9.7)	28 (7.6)	8 (2.2)	22%	< 0.01
60–69 years	14 (4.2)	11 (3.3)	3 (0.9)	21%	0.08
70–79 years	35 (13.9)	26 (10.3)	9 (3.6)	26%	< 0.01
80 + years	28 (18.7)	19 (12.7)	9 (6.0)	32%	< 0.01
Any medicine*					
Total cohort	112 (15.2)	84 (11.4)	28 (3.8)	25%	< 0.001
Males	46 (12.6)	33 (9.1)	13 (3.6)	28%	< 0.001
Females	66 (17.8)	51 (13.7)	15 (4.0)	23%	< 0.001
60–69 years	25 (7.5)	20 (6.0)	5 (1.5)	20%	0.02
70–79 years	46 (18.2)	36 (14.3)	10 (4.0)	22%	< 0.01
80 + years	41 (27.3)	28 (18.7)	13 (8.7)	32%	< 0.001

*Medicines of any type of frequency of administration, including 142 medicines that could not be categorised according to frequency of administration due to missing data

^*p* values calculated using the asymptotic McNamar test

~Relative percentage difference was calculated using active ingredient polypharamcy as the reference

**Table 3:** Characteristics of people with disagreement between polypharmacy exposures according to active ingredient and product count definitions, compared to those with active ingredient polypharmacy

	Scheduled prescription (n = 264)			Scheduled prescriptions and non-prescription medicines (n = 311)			Schedule prescription, non-prescription and CAMs (n = 371)			Any medicine* (n = 421)		
	n (%)	OR (95% CI)	*P* value	n (%)	OR (95% CI)	*P* value	n (%)	OR (95% CI)	*P* value	n (%)	OR (95% CI)	*P* value
Total cohort	41 (15.5)			37 (11.9)			30 (8.1)			33 (7.8)		
Sex												
Females	15 (5.6)	Ref		16 (5.1)	Ref		13 (3.5)	Ref		16 (3.8)	Ref	
Males	26 (9.8)	2.29 (1.15, 4.57)	0.02	21 (6.7)	1.74 (0.87, 3.47)	0.12	17 (4.6)	1.71 (0.8, 3.62)	0.16	17 (4.0)	1.47 (0.72, 2.99)	0.29
Age												
60–69	12 (4.5)	Ref		13 (4.2)	Ref		14 (3.7)	Ref		15 (3.6)	Ref	
70–79	12 (4.5)	0.65 (0.27, 1.53)	0.32	14 (4.5)	0.72 (0.32, 1.63)	0.43	11 (3.0)	0.64 (0.28, 1.46)	0.29	12 (2.8)	0.74 (0.34, 1.65)	0.47
80 +	17 (6.4)	1.35 (0.6, 3.06)	0.47	10 (3.2)	0.69 (0.29, 1.66)	0.40	5 (1.3)	0.39 (0.13, 1.11)	0.08	6 (1.4)	0.49 (0.18, 1.3)	0.15

*Medicines of any type of frequency of administration, including 142 medicines that could not be categorised according to frequency of administration due to missing data

OR, Odds ratios; CAM, Complementary and alternative medicines

Including non-prescription medicines, and then CAMs, in the criteria led to a sequential increase in prevalence estimates. Applying the ‘any medicines’ criteria resulted in an active ingredient prevalence of 57.3%, while product polypharmacy was 52.1% (Table 2). Disagreement between ingredient and product polypharmacy did not appear to be associated with sex or age among the broader inclusion criteria (Table 3). Nevertheless, polypharmacy prevalence estimated across the methods was significantly different between counting methods and remained significant in sex and age stratification (Table 2).

Hyperpolypharmacy was less common with a lower prevalence estimate of 2.3% (≥ 10 prescription unique products), and an upper estimate of 15.2% (≥ 10 active ingredients of any type) (Table 2).

**Fig. 1:**
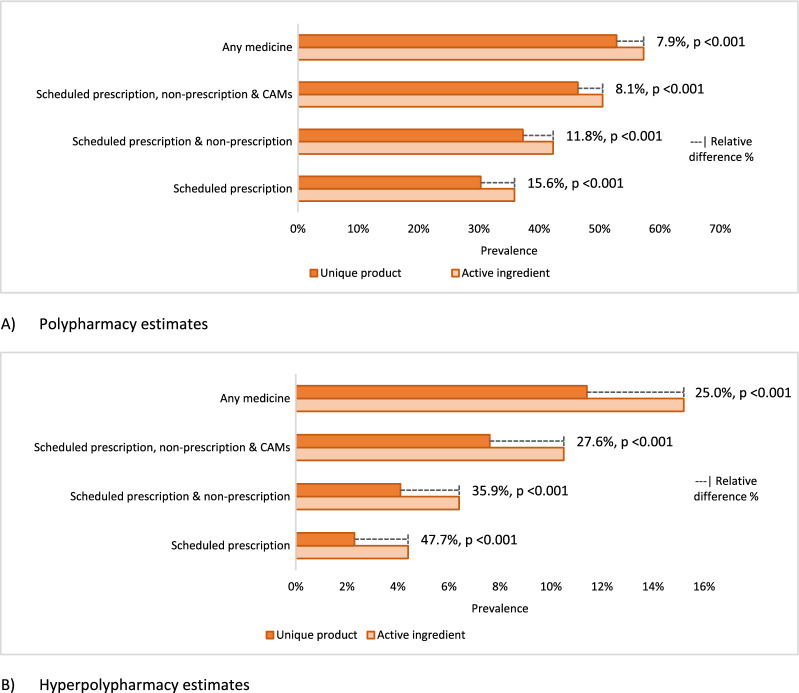
The relative difference between active ingredient and unique product prevalence estimates for **A** Polypharmacy and **B** hyperpolypharmacy by inclusion criteria

Figure 1 shows the absolute and relative percentage difference in prevalence estimates between active ingredient and unique product exposure, according to the method applied. The proportion of people with disagreeing exposure between ingredient and product measures (relative percentage difference) appear to decrease as the inclusion criteria expand; and magnitude of difference appears greater for hyperpolypharmacy (25.0%–47.7%), compared to polypharmacy measures (7.9%–15.6%) (Fig. 1).

## Discussion

### Summary of key findings

Substantial variation in polypharmacy and hyperpolypharmacy prevalence estimates was observed in our sample, depending on the method applied. The proportion exposed to polypharmacy nearly doubled when any medicine by active ingredient counts was compared to unique prescription products, with a 27% variance between upper and lower estimates in the same population. Active ingredient and unique product counting methods were significantly different across both continuous and binary exposure measures. These findings highlight the need for precise descriptions of counting methods and inclusion criteria. Combination products accounted for all discrepancies between the two measures, with more than one in three participants using any combination medicine. The likelihood of combination medicine use was highest among older adults; however, there was no significant association between age and disagreeing active ingredient and unique product polypharmacy exposure. In contrast, men were more likely to use prescription combination products, with 2.3 times the odds of being classified as unexposed to unique product polypharmacy despite using ≥ 5 active ingredients. This suggests that the risk of results misinterpretation may disproportionately affect certain subpopulations when research methods regarding inclusion criteria or counting methods are unclear.

Prevalence estimates varying by method in the same sample have been reported in other population-based studies. In Berlin, polypharmacy rates among community-dwelling older adults (≥ 70 years) increased from 40% with ≥ 5 scheduled prescription medicines to 45% when PRN prescriptions were included [22]. After merging the 5–9 and ≥ 10 medicine categories, prevalence rose to 66% when non-prescription medicines and supplements were counted, reflecting a similar trend in our data. While a prevalence increase with the inclusion additional medicine categories may be expected, it highlights the challenges in interpreting and comparing data when methods are ambiguous. For example, an estimated prevalence of 45.9% was reported for community-dwelling Malaysians aged ≥ 55 years using ≥ 5 prescription, non-prescription and CAMs [23]. Data collection included all medicines used and combination products were counted as a single medicine [23]. However, it is not explicit regarding the inclusion of unscheduled medicines. Consequently, it is unclear if Malaysian prevalence is comparable or lower than our Australian estimates (i.e. 46.5% for scheduled polypharmacy or 52.8% with unscheduled medicines). Evidence also supports our finding of a higher prevalence when using the active ingredient counting method versus unique products [12]. Using dispensing claims data from the Pharmaceutical Benefits Scheme (PBS), the proportion of older adults (≥ 70 years) with ≥ 5 regularly dispensed PBS-subsidised prescription medicines rose from 28.5% to 36.1% when active ingredients were counted individually [12]. This suggests clearly defined counting methods are relevant in both administrative and self-reported medicine data.

The use of combination medicines explained any difference identified between medicine totals according to active ingredient and unique product counts in this study. While the use of combination medicine use may increase the chance of disagreement between polypharmacy exposure, it is expected that disagreement will mainly affect those on the threshold of the cut point applied. Our findings indicate men may be more likely to have a difference in prescription medicine count and polypharmacy exposure status. However, it remains unclear if the risk factors and outcomes of polypharmacy vary for men on the margin of polypharmacy exposure. On the other hand, older adults were no more likely to have disagreement between prescription polypharmacy exposure than their younger counterparts. This null finding was unexpected, given the significantly higher odds of combination product use. However, with only 41 participants identified with disagreeing prescription polypharmacy exposure, this analysis, along with those investigating the more inclusive criteria, was likely underpowered. The current literature on combination medicines appears to focus on cardiovascular disease regimens and clinical trials assessing the impact on adherence and outcome prevention, compared to single-ingredient regimens [24, 25]. This suggests combination use may be most prevalent among those with cardiovascular disease, with potential implications for polypharmacy exposure status in this subgroup. However, there is little evidence on the population-level prevalence and characteristics of combination medicine users to support or refute this finding.

### Strengths and weaknesses

A major strength of this study was the use of self-reported medicine data, allowing for the examination of self-prescribed medicines (non-prescription and CAMs) alongside prescription medicine. This allowed for the investigation of various inclusion criteria by medicine type, in addition to the two methods for measuring polypharmacy in a single, sizable dataset; demonstrating that variability may be attributed to the methods used. Polypharmacy rates in the regions from which the GOS sampling was undertaken (ranged from 37,6360–38,828 to 41,303–42,448 per 100,000 people living in the BSD) appear comparable to the Australian national rate (40,226 per 100,000), as such our prevalence estimates may be more representative of the Australian average, compared to regions with substantially higher or lower rates of medicine utilisation [26].

However, reliance on self-reported data also presents limitations. We did not externally validate the accuracy of reported medicines, so we cannot guarantee their reliability. It is also unclear whether participants interpreted ‘‘current medicines’’ to include unscheduled medicines, potentially resulting in the underreporting of some medicines. During data cleaning, several assumptions were made using the clinical judgment of a pharmacist researcher, which may have introduced bias (i.e. categorising medicines available both with and without a prescription). We did not assess the clinical appropriateness of polypharmacy exposure or relevance of the measures applied. Finally, our analyses of participant characteristics regarding disagreement in polypharmacy exposure may have been underpowered, indicating a need for further investigation.

### Interpretation

Total number of medicines is a variable with utility across a diverse range of research aims. Thus, a single definition for medicine use or polypharmacy is unlikely to be universally appropriate. For example, studies concerned with the risk of suboptimal adherence or medication errors may prioritise methods that count products. When reliable data are available on dose forms, prescribed schedules and administration instructions, tools that also count products, such as the Medicine Regimen Complexity Index (MCRI), may offer a more sensitive calculationby weighting regimens that are more challenging to administer higher than less complex regimens [27]. While these measures provide insight into the administration burden for individuals with polypharmacy, they may be less helpful in identifying therapeutically complex regimens. For research focused on potential harm associated with therapeutic contraindications and interactions, an active ingredient count may be better suited. From a clinical perspective, however, total medicine counts remain only screening tools for potentially suboptimal prescribing; a trigger for comprehensive medication reviews, which should consider both the specific and broader clinical context of the individual.

This research showed there are core aspects to counting medicines that are relevant, regardless of the method applied or dataset used. Clear descriptions of included medicine categories (prescription, non-prescription, CAMs), administration frequency (scheduled, unscheduled), and whether medicines are counted by active ingredient or unique product are essential for interpreting and comparing findings across diverse populations and settings. These findings highlight the need for a minimum reporting standard for medicine counting and defining polypharmacy, which may improve clarity, consistency, and reproducibility of published research. While the clinical significance of more precise methods remains unclear, a reporting standard would provide more robust evidence on polypharmacy prevalence, risk factors and outcomes. Ultimately, these data may better inform and target interventions to reduce preventable medicine-related harm, particularly among older adults.

### Future research

The next step is to conduct a Delphi study with experts to create a reporting checklist of the methods that should be described in research that includes medicine counts. Future research should explore the clinical significance of various polypharmacy methods, and the optimal method for identifying potential underprescribing and high-risk prescribing in-home medication reviews and aged care settings. While further analyses of combination medicine use may also be beneficial.

## Conclusion

In the same study population, polypharmacy prevalence varied by nearly 27% between upper and lower estimates, and by 12.9% for hyperpolypharmacy, depending on the counting method and inclusion criteria used. The difference between active ingredient and unique product counts and polypharmacy exposure also appears to be significant. While polypharmacy methods should remain flexible to be appropriate for specific study aims, settings and populations, consistency in describing the methods used to count and define polypharmacy is essential. A minimum method reporting standard for medicines data is needed to foster greater clarity and reproducibility in polypharmacy research.

## Supplementary Information

Below is the link to the electronic supplementary material.

Supplementary file1 (DOCX 19 KB)

Supplementary file2 (DOCX 18 KB)

Supplementary file3 (DOCX 16 KB)
