# Supplementary material for: Defining polypharmacy in older adults: a cross-sectional comparison of prevalence estimates calculated according to active ingredient and unique product counts
Source: Int J Clin Pharm. 2025 Feb 15;47(3):824–33. doi: 10.1007/s11096-025-01882-7 (PMC12125127; doi:10.1007/s11096-025-01882-7)
Supplement: Supplementary file 1 — Supplementary file1 (DOCX 19 KB) [file 11096_2025_1882_MOESM1_ESM.docx]

| Table S1: Prevalent use of combination medicines by age sex and medicine type | | | | | | | | | | | | |
| --- | --- | --- | --- | --- | --- | --- | --- | --- | --- | --- | --- | --- |
|  | Any combination product* | | | Scheduled medicines | | | | | | | | |
|  |  |  |  | Prescription combination products | | | Non-prescription combination products | | | CAM combination products | | |
|  | n (%) | Univariate OR (95% CI) | P value | n (%) | Univariate OR (95% CI) | P value | n (%) | Univariate OR (95% CI) | P value | n (%) | Univariate OR (95% CI) | P value |
| Total cohort | 278 (37.8) |  |  | 209 (28.4) |  |  | 32 (4.4) |  |  | 30 (4.1) |  |  |
| Sex |  |  |  |  |  |  |  |  |  |  |  |  |
| Females | 130 (35) | ref |  | 84 (22.6) | ref |  | 24 (6.5) | ref |  | 15 (4) | ref |  |
| Males | 148 (40.7) | 1.27 (0.94, 1.71) | 0.12 | 125 (34.3) | 1.79 (1.29, 2.47) | <0.001 | 8 (2.2) | 0.32 (0.14, 0.73) | 0.01 | 15 (4.1) | 1.02 (0.49, 2.12) | 0.96 |
| Age |  |  |  |  |  |  |  |  |  |  |  |  |
| 60-69 | 108 (32.4) | ref |  | 77 (23.1) | ref |  | 9 (2.7) | ref |  | 12 (3.6) | ref |  |
| 70-79 | 99 (39.3) | 1.35 (0.96, 1.9) | 0.09 | 72 (28.6) | 1.33 (0.92, 1.93) | 0.13 | 12 (4.8) | 1.8 (0.75, 4.34) | 0.19 | 12 (4.8) | 1.34 (0.59, 3.03) | 0.49 |
| 80+ | 71 (47.3) | 1.87 (1.26, 2.78) | <0.01 | 60 (40) | 2.22 (1.46, 3.35) | <0.001 | 11 (7.3) | 2.85 (1.15, 7.03) | 0.02 | 6 (4) | 1.11 (0.41, 3.03) | 0.83 |
| * Medicines of any type of frequency of administration, including 142 medicines that could not be categorised according to frequency of administration due to missing data.  CAM – Complementary and alternative medicines  OR – Odds ratio | | | | | | | | | | | | |
