# Supplementary material for: Defining polypharmacy in older adults: a cross-sectional comparison of prevalence estimates calculated according to active ingredient and unique product counts
Source: Int J Clin Pharm. 2025 Feb 15;47(3):824–33. doi: 10.1007/s11096-025-01882-7 (PMC12125127; doi:10.1007/s11096-025-01882-7)
Supplement: Supplementary file 2 — Supplementary file2 (DOCX 18 KB) [file 11096_2025_1882_MOESM2_ESM.docx]

| Table S2: Medicine use per participant according to medicine inclusion criteria (n=735) | | | | | | | | |
| --- | --- | --- | --- | --- | --- | --- | --- | --- |
|  | n (%) | **Active ingredients** | | | **Unique products** | | | **P value^** |
|  |  | Mean (SD) | Median (IQR) | Range  (min, max) | Mean (SD) | Median (IQR) | Range  (min, max) |  |
| **Scheduled medicines** |  |  |  |  |  |  |  |  |
| Prescription medicines | 633 (86.1) | 3.8 (3) | 3 (1, 6) | 20 | 3.5 (2.7) | 3 (1, 5) | 16 | **<.001** |
| Non-prescription medicines | 232 (31.6) | 0.5 (0.8) | 0 (0, 1) | 4 | 0.4 (0.7) | 0 (0, 1) | 4 | **<.001** |
| CAMs | 293 (39.9) | 0.8 (1.4) | 0 (0, 1) | 12 | 0.8 (1.3) | 0 (0, 1) | 12 | **<.001** |
| Prescription and non-prescription medicines | 650 (88.4) | 4.3 (3.2) | 4 (2, 6) | 20 | 3.9 (2.9) | 4 (2, 6) | 19 | **<.001** |
| Prescription and CAMs | 674 (91.7) | 4.6 (3.3) | 4 (2, 7) | 21 | 4.2 (2.9) | 4 (2, 6) | 17 | **<.001** |
| Non-prescription and CAMs | 394 (53.6) | 1.3 (1.7) | 1 (0, 2) | 12 | 1.2 (1.6) | 1 (0, 2) | 12 | **<.001** |
| All scheduled medicines | 679 (92.4) | 5.1 (3.5) | 5 (2, 7) | 21 | 4.6 (3.2) | 4 (2, 7) | 20 | **<.001** |
| **Unscheduled medicines** |  |  |  |  |  |  |  |  |
| PRN medicines | 221 (30.1) | 0.5 (1) | 0 (0, 1) | 9 | 0.5 (0.9) | 0 (0, 1) | 8 | **<.001** |
| Short-course medicines | 16 (2.2) | 0 (0.2) | 0 (0, 0) | 3 | 0 (0.2) | 0 (0, 0) | 2 | .16 |
| **Any medicine*** | 694 (94.4) | 5.8 (4) | 5 (3, 8) | 24 | 5.3 (3.6) | 5 (3, 7) | 22 | **<.001** |
| * Medicines of any type of frequency of administration, including 142 medicines that could not be categorised according to frequency of administration due to missing data.  ^ P values were calculated using Wilcoxon matched-pairs signed-rank test, calculating exact probability; bold p values indicating a significant difference between median active ingredients and median unique products.  CAM – Complimentary or alternative medicines  PRN – *Pro re nata*, medicines taken as needed | | | | | | | | |
