# Supplementary material for: Defining polypharmacy in older adults: a cross-sectional comparison of prevalence estimates calculated according to active ingredient and unique product counts
Source: Int J Clin Pharm. 2025 Feb 15;47(3):824–33. doi: 10.1007/s11096-025-01882-7 (PMC12125127; doi:10.1007/s11096-025-01882-7)
Supplement: Supplementary file 3 — Supplementary file3 (DOCX 16 KB) [file 11096_2025_1882_MOESM3_ESM.docx]

| Table S1: Investigating agreement and disagreement between total active ingredient and product counts according to the prevalent use of combination products | | | |
| --- | --- | --- | --- |
| **Prevalent prescription medicine use (n = 633)** | | | |
|  | Disagreement between total ingredient and product counts | Agreement between total ingredient and product counts | Totals |
| Prevalent combination product use | 424 | 0 | 424 |
| No combination product use | 0 | 209 | 209 |
| Totals | 424 | 209 | Kappa statistic = 1.00, p <0.001 |
| **Prevalent non-prescription medicine use (n = 232)** | | | |
|  | Disagreement between total ingredient and product counts | Agreement between total ingredient and product counts | Totals |
| Prevalent combination product use | 200 | 0 | 200 |
| No combination product use | 0 | 32 | 32 |
| Totals | 200 | 32 | Kappa statistic = 1.00, p <0.001 |
| **Prevalent CAM use (n = 293)** | | | |
|  | Disagreement between total ingredient and product counts | Agreement between total ingredient and product counts | Totals |
| Prevalent combination product use | 263 | 0 | 263 |
| No combination product use | 0 | 30 | 30 |
| Totals | 263 | 30 | Kappa statistic = 1.00, p <0.001 |
| **Prevalent any medicine use (n = 694)** | | | |
|  | Disagreement between total ingredient and product counts | Agreement between total ingredient and product counts | Totals |
| Prevalent combination product use | 416 | 0 | 416 |
| No combination product use | 0 | 278 | 278 |
| Totals | 416 | 278 | Kappa statistic = 1.00, p <0.001 |
| CAM – Complimentary and alternative medicines | | | |
